# Sex-specific genetic effects on susceptibility to idiopathic pulmonary fibrosis

**DOI:** 10.1183/23120541.00200-2025

**Published:** 2025-09-29

**Authors:** Olivia C. Leavy, Anne F. Goemans, Tamara Hernandez-Beeftink, Amy D. Stockwell, Richard J. Allen, Beatriz Guillen-Guio, Ayodeji Adegunsoye, Helen L. Booth, William A. Fahy, Tasha E. Fingerlin, Harvinder S. Virk, Ian P. Hall, Simon P. Hart, Mike R. Hill, Nik Hirani, Naftali Kaminski, Shwu-Fan Ma, Robin J. McAnulty, X. Rebecca Sheng, Ann B. Millar, Maria Molina-Molina, Vidya Navaratnam, Margaret Neighbors, Helen Parfrey, Gauri Saini, Ian Sayers, Mary E. Strek, Martin D. Tobin, Moira K.B. Whyte, Yingze Zhang, Toby M. Maher, Philip L. Molyneaux, Justin M. Oldham, Brian L. Yaspan, Carlos Flores, Fernando Martinez, Carl J. Reynolds, David A. Schwartz, Imre Noth, R. Gisli Jenkins, Louise V. Wain

**Affiliations:** 1 Department of Population Health Sciences, University of Leicester, Leicester, UK; 2 National Institute for Health and Care Research (NIHR) Leicester Biomedical Research Centre, Leicester, UK; 3 Genentech, South San Francisco, CA, USA; 4 University of Chicago, Chicago, IL, USA; 5 University College London Hospitals NHS Foundation Trust, London, UK; 6 Weill Cornell Medicine, New York, NY, USA; 7 GSK, London, UK; 8 National Jewish Health, Denver, CO, USA; 9 University of Nottingham, Nottingham, UK; 10 NIHR Nottingham Biomedical Research Centre, Nottingham, UK; 11 University of Hull, Hull, UK; 12 University of Oxford, Oxford, UK; 13 University of Edinburgh, Edinburgh, UK; 14 Yale School of Medicine, Yale University, New Haven, CT, USA; 15 University of Virginia, Charlottesville, VA, USA; 16 University College London, London, UK; 17 University of Bristol, Bristol, UK; 18 Servei de Pneumologia, Laboratori de Pneumologia Experimental, Instituto de Investigación Biomédica de Bellvitge, Barcelona, Spain; 19 Campus de Bellvitge, Universitat de Barcelona, Barcelona, Spain; 20 Centro de Investigación Biomédica en Red de Enfermedades Respiratorias, Instituto de Salud Carlos III, Madrid, Spain; 21 Department of Respiratory Medicine, Sir Charles Gairdner Hospital, Perth, Australia; 22 Centre for Respiratory Research, University of Western Australia, Perth, Australia; 23 Royal Papworth Hospital NHS Foundation Trust, Cambridge, UK; 24 Centre for Respiratory Research, NIHR Nottingham Biomedical Research Centre, School of Medicine, Biodiscovery Institute, University of Nottingham, Nottingham, UK; 25 University of Pittsburgh, Pittsburgh, PA, USA; 26 NIHR Imperial Biomedical Research Centre, National Heart and Lung Institute, Imperial College London, London, UK; 27 Division of Pulmonary and Critical Care Medicine, University of Southern California, Los Angeles, CA, USA; 28 NIHR Respiratory Clinical Research Facility, Royal Brompton Hospital, London, UK; 29 University of Michigan, Ann Arbor, MI, USA; 30 Research Unit, Hospital Universitario Nuestra Señora de Candelaria, Instituto de Investigación Sanitaria de Canarias, Santa Cruz de Tenerife, Spain; 31 Genomics Division, Instituto Tecnológico y de Energías Renovables, Santa Cruz de Tenerife, Spain; 32 Facultad de Ciencias de la Salud, Universidad Fernando Pessoa-Canarias, Las Palmas de Gran Canaria, Spain; 33 Imperial College London, London, UK; 34 University of Colorado Medicine, Denver, CO, USA; 35 These authors contributed equally

## Abstract

**Background:**

Idiopathic pulmonary fibrosis (IPF) is a chronic lung condition that is more prevalent in males than females. The reasons for this are not fully understood; differing environmental exposures due to historically sex-biased occupations and diagnostic bias are possible explanations. To date, over 20 independent genetic association signals have been reported for IPF susceptibility, but these have been discovered when combining males and females. The objectives of the present study were to assess whether there is a need to consider sex-specific effects when evaluating genetic risk in clinical prediction models for IPF and to test for sex-specific associations with IPF susceptibility.

**Methods:**

We performed a genome-wide single nucleotide polymorphism (SNP)-by-sex interaction study meta-analysis of IPF risk in six independent case–control studies comprising 4561 cases (1280 females, 3281 males) and 22 888 controls (8360 females, 14 528 males) of European genetic ancestry. We used polygenic risk scores (PRSs) comprising common (minor allele frequency >1%) autosomal variants to assess differences in genetic risk prediction between males and females.

**Results:**

The predictive accuracy of the PRSs were similar between males and females, regardless of whether using combined or sex-specific association results. Three new independent genetic association signals were identified (p<1×10^−6^).

**Conclusions:**

The predictive accuracy of common autosomal SNP-based PRSs did not vary significantly between males and females. We prioritised three genetic variants whose effect on IPF risk may be modified by sex. These findings would not account for the differences in prevalence between males and females. Future studies should ensure adequate representation of both sexes.

## Shareable abstract

**Testing for sex-specific associations with IPF identified no evidence for the predictive accuracy of common SNP-based PRSs between men and women** https://bit.ly/4iLu66d

## Introduction

Idiopathic pulmonary fibrosis (IPF) is a progressive fibrotic lung disease with a median survival time after diagnosis of 3–5 years [1]. In the USA and Europe, IPF is estimated to have a disease prevalence of 0.63–7.6 per 100 000 people [2]. The number of people diagnosed with IPF is increasing and males are more likely to be diagnosed than females [3, 4]. However, it is not understood why the disease is more prevalent in males. Different environmental exposures between males and females, notably by occupations such as carpentry, which have traditionally been more common among males [5], could explain some of the observed difference. Diagnostic bias may also play a role, with males being over-diagnosed and females being under-diagnosed with IPF [6]. As well as prevalence differences, there are survival differences, with males having worse survival after IPF diagnosis than females [7]. Differences in genetic predisposition between males and females may be an additional factor in prevalence differences; however, to our knowledge, this has not yet been extensively studied.

IPF is a complex polygenic disease with multiple genes implicated in susceptibility. The genetic variant rs35705950 in the *MUC5B* gene promoter has been shown to increase a person's risk of IPF fivefold for each copy of the risk allele [8–10] and has been estimated to explain more than three times more disease liability than the other known common IPF risk variants combined [11]. In recent years, genome-wide association studies (GWAS), examining genetic variants across the genome, have identified over 20 genetic loci associated with IPF risk [12–15]. In addition to providing new insight into disease biology, polygenic risk scores (PRSs) derived from GWAS data have shown potential utility in identifying individuals at highest risk of pulmonary fibrosis [16], with a recent study demonstrating an area under the receiver operating characteristic (ROC) curve (AUC) of 0.81 for a PRS model combining all common genetic risk factors and clinical data.

Given the increasing interest in the use of PRS as a clinical tool for diagnosis in complex diseases [17], we aimed to test whether PRS for IPF risk performed differently between males and females. We hypothesised that there might be different biological mechanisms that promote IPF susceptibility in males and females and that genetic associations that differ between males and females may pinpoint the genes and pathways involved. To address these questions, we performed a genome-wide single nucleotide polymorphism (SNP)-by-sex interaction meta-analysis of IPF risk in six independent clinically defined IPF case–control studies. We tested for individual genetic variants that might be differentially associated with IPF risk between males and females and that might identify genes that explain observed differences in IPF between males and females.

## Methods

### Studies

The analyses were conducted using six independent IPF case–control studies, all of which have been previously described: US [18] (formerly referred to as Chicago); Colorado [12]; UK [19]; USA, UK and Spain (UUS) [10]; Genentech [20]; and CleanUP-UCD [21, 22] (table 1). In short, unrelated participants from the six studies were included in analyses if they were of genetically determined European ancestry and had sex at birth recorded. We included only participants who passed genotyping quality control, and cases were defined using the relevant American Thoracic Society/European Respiratory Society guidelines [23, 24] (supplementary methods).

**TABLE 1:** Idiopathic pulmonary fibrosis (IPF) case–control cohorts

	Discovery cohorts															Validation cohort	
	US		Colorado		UK		UUS		CleanUP-UCD		Genentech			Total		IPFJES	
**Autosomal data**																	
	Cases	Controls	Cases	Controls	Cases	Controls	Cases	Controls	Cases	Controls	Cases	Controls	Cases	Controls	Total	Cases	Controls
**Males**	374	241	1017	2289	433	2356	597	7210	372	1925	488	507	3281	14 528	17 809	416	2465
**Females**	138	269	498	2394	179	1010	196	2790	93	530	176	1367	1280	8360	9640	NA	NA
**Total**	512	510	1515	4683	612	3366	793	10 000	465	2455	664	1874	4561	22 888	27 449	416	2465
**Chromosome X**																	
			Cases	Controls	Cases	Controls	Cases	Controls	Cases	Controls			Cases	Controls	Total		
**Males**			1017	2289	433	2355	596	7209	376	1925			2422	13 778	16 200		
**Females**			498	2394	179	1010	196	2790	93	530			966	6724	7690		
**Total**			1515	4683	612	3365	792	9999	469	2455			3388	20 502	23 890		

UUS: USA, UK and Spain; IPFJES: Idiopathic Pulmonary Fibrosis Job Exposure Study.

### Polygenic risk score analyses

**FIGURE 1:**
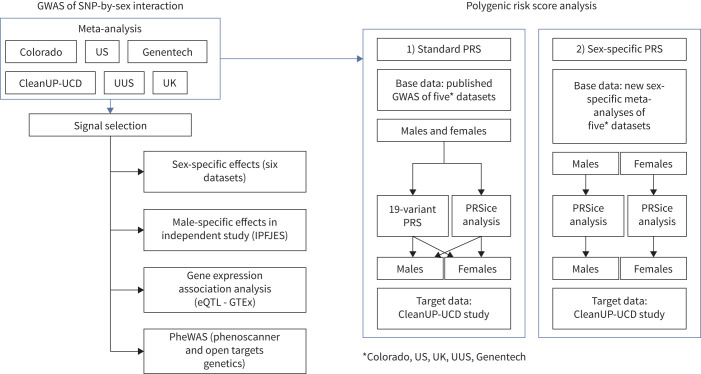
Overview of the single nucleotide polymorphism (SNP)-by-sex interaction analysis and polygenic risk score (PRS) analysis. UUS: USA, UK and Spain; IPFJES: Idiopathic Pulmonary Fibrosis Job Exposure Study; eQTL: expression quantitative trait locus; GTEx: Genotype-Tissue Expression; PheWAS: phenome-wide association studies; GWAS: genome-wide association studies.

We wanted to test whether the predictive accuracy of PRSs in predicting IPF risk differed between males and females. PRSs were constructed by taking the weighted sum of effects across many SNPs and then tested for association with disease risk. The predictive accuracy of a PRS was evaluated in two ways: 1) the predictive performance of the PRS derived from a previously published sex-combined IPF susceptibility GWAS [13] was evaluated in males and females separately (“standard PRS”) and 2) the predictive performance of the PRS derived from sex-specific GWAS was evaluated in males and females separately (“sex-specific PRS”) (figure 1). To do this, we conducted sex-specific GWAS in five of the studies (US, Colorado, UUS, UK and Genentech) using PLINK 1.9 (www.cog-genomics.org/plink/1.9/) and meta-analysed the results (supplementary methods). For 1), we first evaluated a 19-variant PRS representing previously reported common genome-wide significant (p<5×10^−8^) signals of association with IPF [13]. For both 1) and 2), we further incorporated genome-wide data using an approach that varied the threshold used for inclusion of variants in the PRS (PRSice version 2.3.5). “Base data” were derived from the sex-combined and sex-specific autosomal GWAS meta-analyses of the US, Colorado, UK, UUS and Genentech datasets. The “target dataset” was the independent CleanUP-UCD study, comprising 465 cases (93 females and 372 males) and 2455 controls (530 females and 1925 males). AUC differences were tested using the DeLong test (supplementary methods).

### Sex-specific signals of association

Autosomal genome-wide SNP-by-sex interaction analyses of IPF risk were performed separately in all of the six studies described above and meta-analysed using PLINK 1.9 (www.cog-genomics.org/plink/1.9/) (supplementary methods). Chromosome X data were available and analysed for four studies (UUS, Colorado, CleanUP-UCD and UK) (supplementary methods). We took p<5×10^−8^ as the threshold for genome-wide significance and p<1×10^−6^ for suggestive significance. Independent sentinel variants were defined using distance-based and conditional analysis methods (supplementary methods).

As all available datasets with both male and female participants were included within the genome-wide discovery analysis to maximise statistical power, we applied Meta-Analysis Model-based Assessment of replicability (MAMBA) [25] to assess the posterior probability of replication for all SNPs with a meta-analysis p<1×10^−6^. SNPs with a MAMBA posterior probability of replication (PPR) of >90% were considered to be robust across the contributing studies and likely to replicate in future studies.

Male-specific and female-specific effect estimates were calculated for all sentinel variants passing the above criteria. We sought validation of male-specific effect sizes and direction in a male-only IPF case–control study, the IPF Job Exposure Study (IPFJES) [26], comprising 416 male IPF cases and 2465 male controls (figure 1 and supplementary methods). No independent female-only datasets were available at the time of conducting this study, to our knowledge.

Annotation of variants was performed using Variant Effect Predictor (VEP) [27]. We used Genotype-Tissue Expression (GTEx) project data to assess whether or not the sentinel variants were expression quantitative trait loci (eQTLs) for gene expression in up to 49 tissues (including lung and non-lung tissues) and the coloc package in R (version 4.2.1) to test if sentinel variants were eQTLs for gene expression in lung or cultured fibroblasts (figure 1 and supplementary methods). We conducted phenome-wide association studies (PheWAS) using PhenoScanner [28] and Open Targets (www.opentargets.org) to examine whether the signals were also associated with other phenotypes.

## Results

### Polygenic risk scores

**FIGURE 2:**
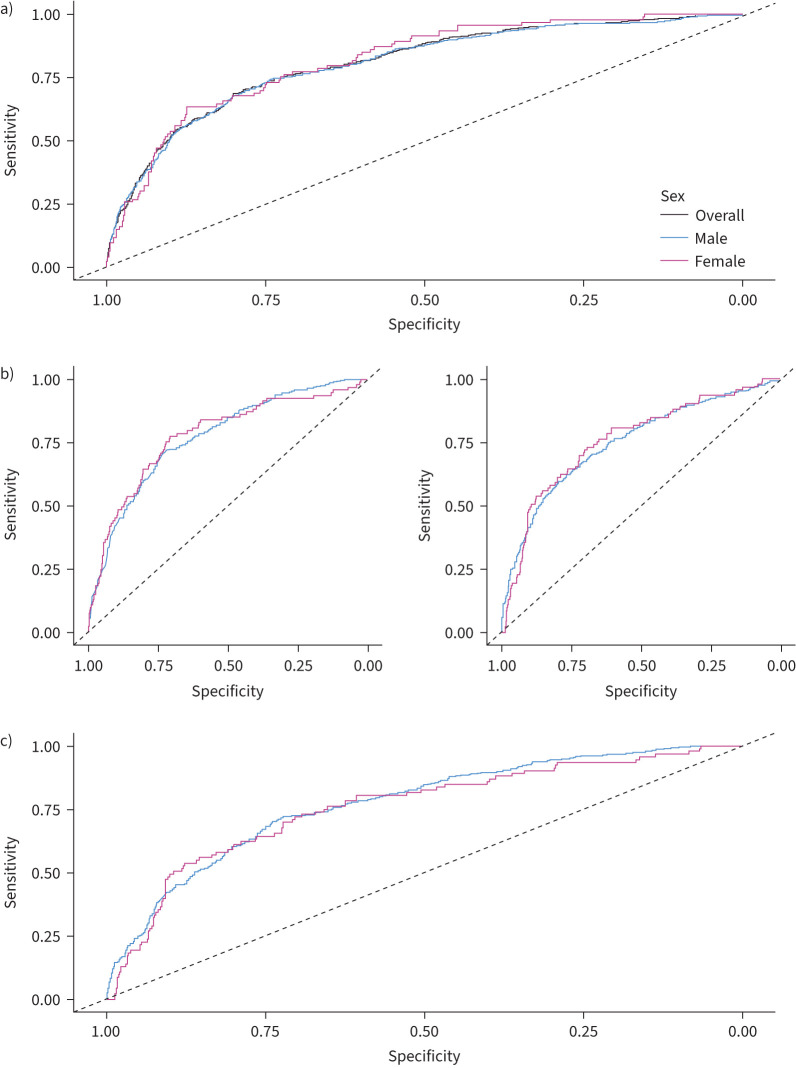
Summary of results area under the curve (AUC) for prediction in polygenic risk score (RPS) analysis in a) PRS derived from standard genome-wide association studies (GWAS) overall and applied to males and females; b) PRS derived from male-specific GWAS applied to males and females (left), and PRS derived from female-specific GWAS applied to males and females (right); and c) PRS from male-specific GWAS applied to males *versus* PRS from female-specific GWAS applied to females.

For the “standard PRS” analysis, there was no difference in the AUC between males and females for the 19-variant PRS (AUC males: 80.3% *versus* AUC females: 80.8%, DeLong p=0.85) (supplementary table 1 and figure 2). When constructing multiple PRSs (*i.e.* not limiting to published IPF susceptibility variants), the most predictive p-value threshold (*P*_T_) was *P*_T_<4.5×10^−4^ for males and *P*_T_<5×10^−4^ for females (supplementary figure 1a, b), and while the AUC estimated was slightly lower for males than for females, the difference was not statistically significant (male AUC: 80.2% *versus* female AUC: 81.8%, DeLong p=0.54).

The sex-specific GWAS were performed in up to 1187 female cases and 6970 female controls, and 2909 male cases and 12 603 male controls. For the “sex-specific PRS” analysis, the male-specific PRS predictive accuracy was slightly higher than the female-specific PRS predictive accuracy, but the difference was not statistically significant (male-specific PRS AUC: 78.2% *versus* female-specific PRS AUC: 76%, DeLong p=0.47) (supplementary table 1 and supplementary figure 1c, d). The AUCs observed in this analysis were smaller than those generated in the “standard PRS” analysis, which might be explained by the decrease in sample size of the training set (*i.e.* less accurate effect sizes).

The PRS results suggest that the predictive accuracy of IPF PRSs is not statistically different between males and females, regardless of whether combined or sex-specific GWAS results are used.

### Sex-specific signals

**FIGURE 3:**
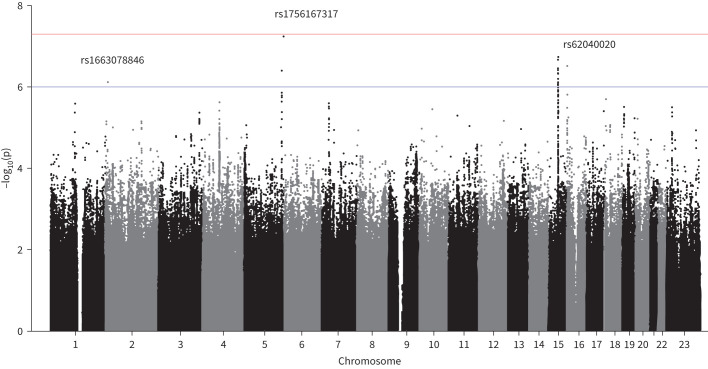
Manhattan plot of meta-analysed sex-interaction results. The chromosomal position is on the x-axis and the −log(p-value) for each genetic variant in the sex-interaction meta-analysis is on the y-axis. Variants present in at least three studies are presented. The blue horizontal line represents the 1×10^−6^ p-value threshold and the red horizontal line represents the 5×10^−8^ p-value threshold (genome-wide significance threshold).

The autosomal genome-wide sex interaction analysis of IPF risk was performed in up to 4561 cases (comprising 1280 females and 3281 males) and 22 888 controls (8360 females and 14 528 males) (table 1). For chromosome X, sex-interaction analyses were performed for 3388 IPF cases (966 females and 2422 males) and 20 502 controls (6724 females and 13 778 males). There was no evidence of inflated test statistics (figure 3 and supplementary figure 2).

**FIGURE 4:**
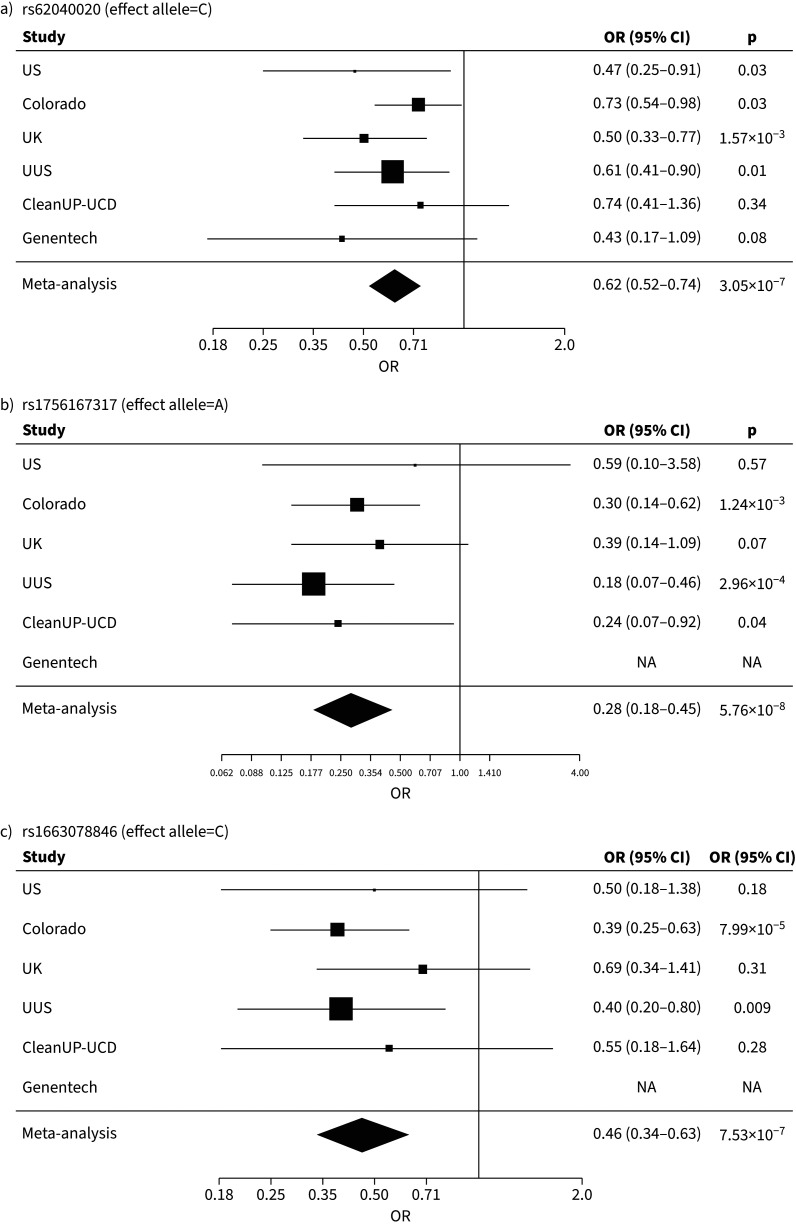
Forest plots showing single nucleotide polymorphism (SNP)–sex interaction odds ratio (OR) by study and the meta-analysed results for a) rs62040020, b) rs1756167317 and c) rs1663078846. CI: confidence interval; UUS: USA, UK and Spain.

In the autosomal analysis, three independent sentinel variants with interaction p<1×10^−6^ and MAMBA PPR>90% were identified (supplementary tables 2 and 3). All three variants had consistent direction of effect across all contributing studies (figure 4a, b, c). One signal was close genome-wide significance (rs1756167317, p=5.76×10^−8^). No signals reached the predefined significance thresholds for the chromosome X analysis.

**FIGURE 5:**
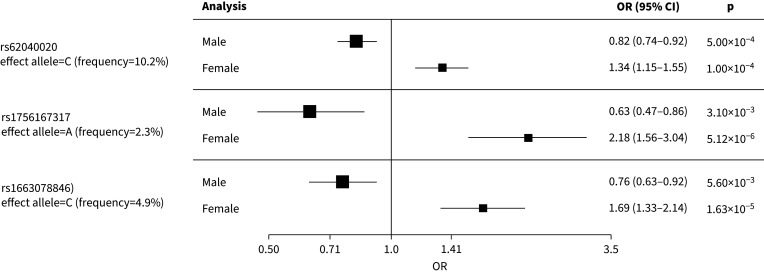
Forest plot for sex-stratified results meta-analysed across US, Colorado, UK, USA, UK and Spain (UUS), CleanUP-UCD and Genentech. OR: odds ratio; CI: confidence interval.

The sentinel variant rs62040020, which resided within an intron of Jupiter microtubule associated homolog 2 (*JPT2*) on chromosome 16 (effect allele frequency (EAF)=10.6%), was measured in all six studies with a high imputation quality (r^2^>0.88 across all six studies) and was nominally significant (p<0.05) in four of the six studies (supplementary table 2 and figure 4). When tested for association with IPF risk in females and males separately, the minor allele (allele=C) of rs62040020 was associated with increased risk of IPF in females (odds ratio (OR) 1.34, 95% confidence interval (CI) 1.15–1.55; p=1×10^−4^) and a decreased risk in males (OR 0.82, 95% CI 0.74–0.92, p=1×10^−3^) (figure 5). Accordingly, if we instead took the alternative allele to be the effect allele (allele=G), the direction of effect would be in the opposite direction (*i.e.* increased risk of IPF in males and decreased risk in females). In the male-only IPFJES, the association effect was close to the null and nonsignificant (OR 0.99, 95% CI 0.77–1.27, p=0.939) (supplementary figure 4a). In lung and/or cultured fibroblasts, the C allele of rs62040020 (associated with increased IPF risk in females and decreased risk in males) was associated with increased expression of fumarylacetoacetate hydrolase domain containing 1 (*FAHD1*), meiosis specific with OB-fold (*MEIOB*) and NUBP iron-sulfur cluster assembly factor 2, cytosolic (*NUBP2*), and decreased expression of mitochondrial ribosomal protein S34 (*MRPS34*) (supplementary tables 4 and 5). This allele was also related to changes in splicing of hydroxyacylglutathione hydrolase (*HAGH*) in lung, cultured fibroblasts and a range of other tissues. However, rs62040020 was not the most significant variant associated with expression of these genes at this locus, with co-localisation analyses for male sex-specific GWAS results suggesting the GWAS and eQTL association signals were driven by different variants (supplementary figure 5, supplementary tables 4 and 5 and supplementary methods). The C allele of this variant was previously nominally associated with reduced monocyte percentage in UK Biobank (NEALE round 2 results: www.nealelab.is/uk-biobank/; p=1.7×10^−4^) (supplementary table 4).

For the other two SNPs, rs1756167317 and rs1663078846, the sentinel variants were of low frequency (EAF: 1–5%) and were nominally significant in three out of five contributing studies and two out of five contributing studies, respectively (figure 4b, c and supplementary table 2). The A allele of rs1756167317, located in an intron of proline rich 7, synaptic (*PRR7*) at chromosome 5 (supplementary figure 3b), was associated with increased risk of IPF in females (OR 2.18, 95% CI 1.56–3.04; p=5×10^−6^) and decreased risk in males (OR 0.63, 95% CI 0.47–0.86; p=3×10^−3^) (figure 5). This SNP showed a consistent direction and size of effect in the male-only IPFJES (OR 0.68, 95% CI 0.36–1.30; p=0.247) (supplementary figure 4b). For the sentinel variant of the signal on chromosome 2, rs1663078846, the C allele was associated with increased risk of IPF in females (OR 1.69, 95% CI 1.33–2.14; p=2×10^−5^) and decreased risk in males (OR 0.76, 95% CI 0.63–0.92; p=6×10^−3^) (figure 5). This intergenic variant (supplementary figure 3c) did not show a consistent effect in males in IPFJES (OR 1.07, 95% CI 0.74–1.55; p=0.702) (supplementary figure 4c). No associations with other traits for rs1756167317 or rs1663078846 were found in PhenoScanner or Open Targets.

None of the common previously reported IPF susceptibility variants, including the *MUC5B* promoter variant rs35705950, were observed to have a significant sex interaction effect when accounting for multiple testing (supplementary table 6), although the desmoplakin (*DSP*) variant, rs2076295, had a nominally significant interaction effect (p=0.03) (supplementary figure 6).

## Discussion

We performed the first, to our knowledge, genome-wide SNP-by-sex interaction analysis of IPF risk in clinically defined cases. We found that the predictive accuracies of PRSs based on common autosomal variants were not sex dependent, suggesting that PRSs developed from sex-combined association statistics are largely generalisable across sexes. Although we highlight three new suggestively significant signals with differential effects on IPF risk by sex, overall, our study suggests that observed differences in prevalence of IPF between males and females are not explained by common autosomal genetic effects.

None of the 19 previously reported common IPF genetic variants [13] demonstrated a significant sex interaction (p<1×10^−6^). Previously, a sex-stratified meta-analysis conducted across six biobank studies [14] observed a larger effect size in males than in females for the *MUC5B* variant rs35705950. The effect was not replicated in a clinically defined IPF sub-study of FinnGen or in the four clinically defined case–control studies (four of the six studies used were included in our present study). We also did not observe this difference in our analysis (which included additional datasets)*.* We have previously highlighted differences in genetic association effect sizes when defining IPF from routine electronic healthcare records, compared with clinically defined cohorts [29], suggesting that case definition heterogeneity might account for the biobank finding.

Although the three new signals identified here were consistent across all studies included in the meta-analysis, none of them replicated in the male-only IPFJES; this could have been because the study was under-powered to validate the male-specific effects. Further data are needed to provide confidence in these signals and to confirm the probable causal genes at these loci. These new signals, however, may offer further insight into sex-specific mechanisms in IPF. *FAHD1*, *HAGH* and *MRPS34* have all been implicated in mitochondrial function. Mitochondrial dysfunction has been widely implicated in age-related disease such as IPF [30]. *HAGH* was also amongst 2940 genes differentially expressed between IPF cases and controls [31]. However, the sex-specific effect of these genes has not been investigated.

Studies of interaction effects require larger sample sizes than GWAS of main effects on disease risk as we are testing for a difference in effect size between two subgroups of participants. We cannot exclude the possibility that there are additional sex-specific genetic association signals yet to be discovered with larger sample sizes. However, our PRS analysis also included variants not meeting stringent statistical significance and did not suggest that there were any large effect sex-specific signals yet to be detected. There were more males than females in the analysis, which would affect the 95% CI of the AUC, with females having a wider AUC 95% CI than males.

Our study has some limitations. It may be that sex differences might be observed in mitochondrial DNA and/or the Y chromosome, but we do not have those data available. There may also be a bias in diagnostic assignment based on sex, particularly in the classification of females as having non-IPF interstitial lung disease (ILD) phenotypes, and we cannot address the possibility of a sex-interaction effect for ILD subtypes in females. Our PRS analysis was not intended to define the optimum PRS for IPF in either or both sexes, but rather to indicate whether future efforts should focus on sex-specific PRS development. As our data were based on array genotyping with imputation, our study was limited to variants with minor allele frequency (MAF) >1%; as such, we cannot exclude the potential for rare variants with large sex-specific effects. All data included in this study were derived from individuals of European ancestry. Thus, our findings may not be generalisable to other ancestries, and larger genetic studies of ILD in non-European ancestry populations, with appropriate representation of both sexes, are urgently needed.

In summary, our PRS analysis suggests that PRS derived from sex-combined IPF SNP association studies perform similarly in males and females with no significant benefit in deriving sex-specific PRS. We identified three potential sex-interaction signals which require further validation and functional investigation. In conclusion, our study suggests that common (MAF>1%) autosomal genetic variation does not account for the differences in prevalence of IPF between males and females.

## Data Availability

The summary statistics for the genome-wide SNP–sex interaction meta-analysis can be accessed from https://github.com/genomicsITER/PFgenetics.

## Supplementary material

**Please note:** supplementary material is not edited by the Editorial Office, and is uploaded as it has been supplied by the author. Supplementary material 00200-2025.SUPPLEMENT Table S2 00200-2025.SUPPLEMENT2 Table S3 00200-2025.SUPPLEMENT3 Table S4 00200-2025.SUPPLEMENT4 Table S5 00200-2025.SUPPLEMENT5 Table S6 00200-2025.SUPPLEMENT6 Table S1 00200-2025.SUPPLEMENT7
