# Supplementary material for: Sex-specific genetic effects on susceptibility to idiopathic pulmonary fibrosis
Source: ERJ Open Res. 2025 Sep 29;11(5):00200-2025. doi: 10.1183/23120541.00200-2025 (PMC12477485; doi:10.1183/23120541.00200-2025)
Supplement: Supplementary file 3 [file 00200-2025.SUPPLEMENT3.pdf]

Table S3: Sex-specific allele frequencies in cases vs. control for the sentinels variants that reach P<1×10-6 and posterior probability of replication (MAMBA) >90%

SNP	Sex	**US** (case/control)	**Colorado** (case/control)	**UK** (case/control)	**UUS** (case/control)	**Genentech** (case/control)	**CleanUP-UCD** (case/control)
rs1663078846	Male	0.069/0.094	0.029/0.023	0.027/0.027	0.022/0.025	NA	0.015/0.029
	Female	0.087/0.058	0.057/0.019	0.046/0.027	0.040/0.023	NA	0.037/0.033
rs1756167317	Male	0.010/0.009	0.010/0.010	0.011/0.012	0.006/0.012	NA	0.007/0.010
	Female	0.004/0.004	0.020/0.007	0.024/0.008	0.028/0.012	NA	0.034/0.012
rs62040020	Male	0.017/0.034	0.082/0.089	0.081/0.111	0.085/0.106	0.078/0.098	0.090/0.102
	Female	0.042/0.042	0.106/0.082	0.134/0.101	0.117/0.101	0.082/0.089	0.118/0.106
