# Supplementary material for: Sex-specific genetic effects on susceptibility to idiopathic pulmonary fibrosis
Source: ERJ Open Res. 2025 Sep 29;11(5):00200-2025. doi: 10.1183/23120541.00200-2025 (PMC12477485; doi:10.1183/23120541.00200-2025)
Supplement: Supplementary file 4 [file 00200-2025.SUPPLEMENT4.pdf]

Table S4: Annotation and eQTL results for variants in 95% credible set of IPF SNP-by-sex signals

																		Gtex									PheWAS		HaploReg				
																		Lung			Cultured fibroblasts			Other tissues									
CHR	Position (Build38)	rsid	Effect allele	Other allele	N studies	P	OR	OR lower 95%CI	OR upper 95%CI	beta	se	z	Posterior probability	Cumulative sum	Note	Annotation (VEP)	GWAS Catalog	Gene	Normalised effect size	p-value	Gene	Normalised effect size	p-value	Gene	p-value	Tissue	Open Targets	PhenoScanner	Promoter histone marks	Enhancer histone marks	DNAse	Motifs changed	NHGRI/EBI GWAS hits
2	11000268	rs1663078846	C	T	5	7.53E-07	0.46	0.34	0.63	-0.777	0.157	-4.947	100.0%	1	Sentinel	Intergenic	X											X					
5	177451510	rs1756167317	A	C	5	5.76E-08	0.28	0.18	0.45	-1.256	0.232	-5.426	100.0%	1	Sentinel	Intron of *PRR7*	X											X					
16	1697584	rs62040020	C	G	6	3.05E-07	0.62	0.52	0.74	-0.479	0.093	-5.120	88.4%	0.884	Sentinel	Intron of *JPT2*	X	*FAHD1*	0.43	4.90E-23	*MEIOB*	-0.51	9.40E-09	Lots of results in other tissues			smallest p = 0.00017, Monocyte percentage (beta = -0.0395)	Non-applicable results (best p = x10-4)	X	PLCNT	X	7 altered motifs	X
																		HAGH	0.20	3.00E-06	*FAHD1*	0.31	8.50E-08										
																		MEIOB	0.41	8.60E-06	*MRPS34*	-0.09	4.30E-05										
																					HAGH	0.18	6.60E-05										
																					NUBP2	0.11	9.30E-05										
16	1581293	rs56218869	G	A	6	2.09E-05	0.63	0.51	0.78	-0.466	0.109	-4.255	2.3%	0.907		Intron of *IFT140*	X	*FAHD1*	0.31	2.10E-08	X		X	Lots of results in other tissues			smallest p = 0.00016, Bursitis of shoulder (beta = 0.551)		X	GI, LIV	X	Hoxa5	X
16	1566527	rs116908844	A	T	6	2.39E-05	0.63	0.51	0.78	-0.464	0.110	-4.225	2.0%	0.927		Intron of *IFT140*	X	*FAHD1*	0.32	3.30E-09	X		X	Lots of results in other tissues			smallest p = 0.00020, Bursitis of shoulder (beta = 0.541)		X	X	X	X	X
16	1753892	rs117959643	A	G	6	7.89E-06	0.44	0.30	0.63	-0.832	0.186	-4.468	0.6%	0.933		Intron of *MAPK8IP3*	Insulin-like growth factor 1 levels (risk allele = A, beta = 0.0795, P = 9x10-21	X		X	X		X	*EME2*	1.1E-06	Skin - Not Sun Exposed (Suprapubic)	p = 8.6e-38, Insulin-like growth factor 1 levels (beta = 0.0822)		X	X	X	Hic1	X
																								IGFALS	3.9E-06	Nerve - Tibial							
16	1869176	rs184850749	A	G	6	1.55E-06	0.35	0.23	0.53	-1.058	0.220	-4.805	0.6%	0.939		Intron of *MEIOB*	X	X		X	X		X				smallest p = 0.000012, Basophil percentage of white cells (beta = 0.0421)		X	X	LIV	ERE,TEF-1,Zbtb3	X
16	1776040	rs190542947	A	G	6	3.22E-06	0.38	0.25	0.57	-0.979	0.210	-4.656	0.6%	0.945		Intron of *EME2*	X	X		X	X		X				p = 6.7e-31, Insulin-like growth factor 1 levels (beta = 0.0836)		X	10 tissues	5 tissues	5 altered motifs	X
16	1605758	rs117492052	A	G	6	1.03E-05	0.40	0.27	0.60	-0.912	0.207	-4.412	0.4%	0.948		Intron of *IFT140*	X	X		X	X		X	*EME2*	4.80E-05	Skin - Not Sun Exposed (Suprapubic)	smallest p = 0.0000058, Standing height (beta=-0.248), next smallest p = 0.000036 Fev1 predicted		X	X	X	GATA,Mef2,Obox	X
16	1776667	rs191479216	C	T	6	1.16E-05	0.40	0.27	0.61	-0.906	0.207	-4.384	0.3%	0.952		3 prime UTR variant (*EME2*)	X	X		X	X		X	X	X	X	smallest p = 0.000016, Standing height (beta=-0.241)		X	7 tissues	8 tissues	HEY1,Hic1,YY1	X

Note: CHR = chromosome. N= number. OR = odds ratio. 95% CI = 95% confidence interval. se = standard error. VEP = Ensembl Variant Effect Predictor
