# Supplementary material for: Sex-specific genetic effects on susceptibility to idiopathic pulmonary fibrosis
Source: ERJ Open Res. 2025 Sep 29;11(5):00200-2025. doi: 10.1183/23120541.00200-2025 (PMC12477485; doi:10.1183/23120541.00200-2025)
Supplement: Supplementary file 5 [file 00200-2025.SUPPLEMENT5.pdf]

Table S5: Results of colocalisation analysis between rs62040020 and lung tissue and cultured fibroblasts using Coloc (female and male specific results)

SNP of interest: rs62040020 (CHR16: 1697584)

Female

CHR	Lower position (Build38)	Upper position (Build38)	GWAS N	Tissue (GTEx)	Gene	EQTL N	nSNPs	H0	H1	H2	H3	H4
16	697584	2697584	9640	Lung	*HAGH*	515	6160	1.58E-30	2.01E-30	0.42	0.54	0.04
16	697584	2697584	9640	Lung	*MEIOB*	515	5954	5.29E-03	6.69E-03	0.42	0.53	0.03
16	697584	2697584	9640	Lung	*FAHD1*	515	6156	2.02E-62	2.58E-62	0.43	0.55	0.02
16	697584	2697584	9640	Cultured fibroblasts	*HAGH*	483	6160	4.73E-12	6.02E-12	0.43	0.55	0.02
16	697584	2697584	9640	Cultured fibroblasts	*MEIOB*	483	5954	1.09E-15	1.38E-15	0.43	0.55	0.02
16	697584	2697584	9640	Cultured fibroblasts	*FAHD1*	483	6156	8.05E-45	1.02E-44	0.43	0.55	0.02
16	697584	2697584	9640	Cultured fibroblasts	*MRPS34*	483	6408	6.03E-20	7.76E-20	0.41	0.52	0.07
16	697584	2697584	9640	Cultured fibroblasts	*NUBP2*	483	6375	5.35E-02	6.88E-02	0.38	0.48	0.02

Male

CHR	Lower position (Build38)	Upper position (Build38)	GWAS N	Tissue (GTEx)	Gene	EQTL N	nSNPs	H0	H1	H2	H3	H4
16	697584	2697584	17809	Lung	*HAGH*	515	6158	5.22E-31	3.20E-30	0.14	0.86	2.13E-03
16	697584	2697584	17809	Lung	*MEIOB*	515	5952	1.73E-03	0.01	0.14	0.84	0.01
16	697584	2697584	17809	Lung	*FAHD1*	515	6154	6.56E-63	4.02E-62	0.14	0.86	3.60E-03
16	697584	2697584	17809	Cultured fibroblasts	*HAGH*	483	6158	1.54E-12	9.41E-12	0.14	0.86	3.19E-03
16	697584	2697584	17809	Cultured fibroblasts	*MEIOB*	483	5952	3.54E-16	2.16E-15	0.14	0.86	3.94E-03
16	697584	2697584	17809	Cultured fibroblasts	*FAHD1*	483	6154	2.62E-45	1.60E-44	0.14	0.86	2.68E-03
16	697584	2697584	17809	Cultured fibroblasts	*MRPS34*	483	6406	2.07E-20	1.27E-19	0.14	0.86	2.79E-03
16	697584	2697584	17809	Cultured fibroblasts	*NUBP2*	483	6373	0.02	0.11	0.12	0.74	0.02

Note: CHR = chromosome, N= number, GWAS = genome-wide association study, EQTL = expression quantitative trait loci, SNP = single nucleotide polymorphism
