# Supplementary material for: Sex-specific genetic effects on susceptibility to idiopathic pulmonary fibrosis
Source: ERJ Open Res. 2025 Sep 29;11(5):00200-2025. doi: 10.1183/23120541.00200-2025 (PMC12477485; doi:10.1183/23120541.00200-2025)
Supplement: Supplementary file 6 [file 00200-2025.SUPPLEMENT6.pdf]

**Table S6: SNP-by-sex interaction meta-analysis results for previously reported IPF susceptibility SNPs**

| rsid | CHR | Position (Build38) | Effect allele | Other allele | N | *P* | OR | Proxy SNP | CHR | Position (Build38) | Effect allele | Other allele | N | *P* | OR |
|---|---|---|---|---|---|---|---|---|---|---|---|---|---|---|---|
| rs2292181 | 3 | 44861942 | C | G | 6 | 0.558 | 0.93 | NA | NA | NA | NA | NA | NA | NA | NA |
| rs9860874 | 3 | 169768483 | A | C | 6 | 0.601 | 0.97 | NA | NA | NA | NA | NA | NA | NA | NA |
| rs2609259 | 4 | 88916657 | A | C | 6 | 0.763 | 1.02 | NA | NA | NA | NA | NA | NA | NA | NA |
| rs7725218 | 5 | 1282299 | A | G | 6 | 0.251 | 0.93 | NA | NA | NA | NA | NA | NA | NA | NA |
| rs2076295 | 6 | 7562999 | G | T | 6 | 0.028 | 1.13 | NA | NA | NA | NA | NA | NA | NA | NA |
| rs12537430 | 7 | 1829125 | A | G | 6 | 0.479 | 1.04 | NA | NA | NA | NA | NA | NA | NA | NA |
| rs2897075 | 7 | 100032719 | T | C | 6 | 0.954 | 1.00 | NA | NA | NA | NA | NA | NA | NA | NA |
| rs10808505 | 8 | 119927966 | G | T | 6 | 0.221 | 0.93 | NA | NA | NA | NA | NA | NA | NA | NA |
| rs79684490 | 10 | 109470103 | A | G | 6 | 0.260 | 0.87 | NA | NA | NA | NA | NA | NA | NA | NA |
| rs35705950 | 11 | 1219991 | T | G | 6 | 0.748 | 1.03 | NA | NA | NA | NA | NA | NA | NA | NA |
| rs9577395 | 13 | 112880670 | G | C | 6 | 0.461 | 0.95 | NA | NA | NA | NA | NA | NA | NA | NA |
| rs2304645 | 15 | 40424054 | G | C | 6 | 0.327 | 1.06 | NA | NA | NA | NA | NA | NA | NA | NA |
| rs12912339 | 15 | 40639510 | A | G | 6 | 0.426 | 0.94 | NA | NA | NA | NA | NA | NA | NA | NA |
| rs11073517 | 15 | 85744679 | T | C | 6 | 0.269 | 1.07 | NA | NA | NA | NA | NA | NA | NA | NA |
| rs74614704 | 16 | 112241 | A | G | 6 | 0.645 | 0.95 | NA | NA | NA | NA | NA | NA | NA | NA |
| rs2077551 | 17 | 46137522 | NA | NA | NA | NA | NA | rs17652520 | 17 | 46021601 | A | G | 5 | 0.566 | 0.9576 |
| rs12610495 | 19 | 4717660 | G | A | 6 | 0.638 | 0.97 | NA | NA | NA | NA | NA | NA | NA | NA |
| rs112087793 | 20 | 63652817 | T | C | 6 | 0.658 | 1.05 | NA | NA | NA | NA | NA | NA | NA | NA |
| rs41308092 | 20 | 63693038 | A | G | 6 | 0.162 | 0.78 | NA | NA | NA | NA | NA | NA | NA | NA |

*Note: rs17652520 was used as a proxy for rs2077551*

CHR = chromosome,   N= number, OR = odds ratio
