# Supplementary material for: Sex-specific genetic effects on susceptibility to idiopathic pulmonary fibrosis
Source: ERJ Open Res. 2025 Sep 29;11(5):00200-2025. doi: 10.1183/23120541.00200-2025 (PMC12477485; doi:10.1183/23120541.00200-2025)
Supplement: Supplementary file 7 [file 00200-2025.SUPPLEMENT7.pdf]

Table S1: Polygenic risk score analyses results i) 'standard PRS' analysis ii) 'sex-specific PRS' analysis

i) 'standard PRS' analysis

								DeLong's test for two ROC curves		
**Training**	**Target**	**Target population**	**SNP *p* -value threshold**	**nSNPs**	***p* -value**	**AUC (%)**	**95% CI (%)**	D	df	*p* -value
Allen *et al* 2022	CleanUP-IPF	Overall	Published genome-wide sig	19	1.96x10-145	80.3	[78.1, 82.5]			
		Males			2.94x10-117	80.3	[77.8, 82.7]	-0.2	907.56	0.85
		Females			1.87x10-30	80.8	[75.6, 86.1]			
P-value thresholding										
**Training**	**Target**	**Target population**	**Best SNP *p* -value threshold**	**nSNPs**	***p* -value**	**AUC (%)**	**95% CI (%)**			
Allen *et al* 2022	CleanUP-IPF	Overall	4.50E-04	1028	2.73x10-83	80.5	[78.3, 82.7]			
		Males	4.50E-04	1027	3.70x10-66	80.2	[77.7, 82.7]	-0.6155	1053.5	0.54
		Females	5.00E-04	1098	1.42x10-18	81.8	[77.4, 86.3]			

ii) 'sex-specific PRS' analysis

**Training**	**Target**	**Target population**	**Best SNP *p* -value threshold**	**nSNPs**	***p* -value**	**AUC (%)**	**95% CI (%)**
Male specific (Colorado, US, UUS, UK, Genentech)	CleanUP-IPF	Males	0.3765	249644	6.00E-58	78.2	[75.7, 80.8]
		Females	5.01E-05	453	2.39E-17	78.7	[73.3, 84.1]
Female specific (Colorado, US, UUS, UK, Genentech)	CleanUP-IPF	Males	5.00E-08	28	1.54E-53	74.9	[72.0, 77.8]
		Females	5.000E-08	28	2.99E-14	76.0	[70.4, 81.5]

DeLong's test for two ROC curves

	AUCs	D	df	*p* -value
male-male vs female-female	78.2% vs 76%	0.7188	901.09	0.47

Note: PRS = polygenic risk score, SNP = single nucleotide polymorphism, ROC curve = receiver operating characteristic curve, AUC = area under the ROC Curve, df = degrees of freedom, 95% CI = 95% confidence interval
